# SNAP-25 phosphorylation at Ser187 regulates synaptic facilitation and short-term plasticity in an age-dependent manner

**DOI:** 10.1038/s41598-017-08237-x

**Published:** 2017-08-11

**Authors:** Norikazu Katayama, Saori Yamamori, Masahiro Fukaya, Shizuka Kobayashi, Masahiko Watanabe, Masami Takahashi, Toshiya Manabe

**Affiliations:** 1 Division of Neuronal Network, Department of Basic Medical Sciences, Institute of Medical Science, University of Tokyo, Tokyo, 108-8639 Japan; 2 Department of Biochemistry, Kitasato University School of Medicine, Sagamihara, 252-0374 Japan; 3 Department of Anatomy, Kitasato University School of Medicine, Sagamihara, 252-0374 Japan; 4 Department of Anatomy, Hokkaido University Graduate School of Medicine, Sapporo, 060-8638 Japan

## Abstract

Neurotransmitter release is mediated by the SNARE complex, but the role of its phosphorylation has scarcely been elucidated. Although PKC activators are known to facilitate synaptic transmission, there has been a heated debate on whether PKC mediates facilitation of neurotransmitter release through phosphorylation. One of the SNARE proteins, SNAP-25, is phosphorylated at the residue serine-187 by PKC, but its physiological significance has been unclear. To examine these issues, we analyzed mutant mice lacking the phosphorylation of SNAP-25 serine-187 and found that they exhibited reduced release probability and enhanced presynaptic short-term plasticity, suggesting that not only the release process, but also the dynamics of synaptic vesicles was regulated by the phosphorylation. Furthermore, it has been known that the release probability changes with development, but the precise mechanism has been unclear, and we found that developmental changes in release probability of neurotransmitters were regulated by the phosphorylation. These results indicate that SNAP-25 phosphorylation developmentally facilitates neurotransmitter release but strongly inhibits presynaptic short-term plasticity via modification of the dynamics of synaptic vesicles in presynaptic terminals.

## Introduction

Excitatory synaptic transmission in the central nervous system (CNS) is mediated by the neurotransmitter glutamate, which is released from synaptic vesicles in the presynaptic terminal through membrane fusion of the terminal and synaptic vesicles in a Ca^2+^-dependent manner^1^, and the released glutamate binds to postsynaptic glutamate receptors and transmits neural information to postsynaptic cells. The SNARE proteins, including the synaptic vesicle SNARE synaptobrevin and the plasma membrane SNAREs syntaxin and SNAP-25, play crucial roles in exocytosis of synaptic vesicles for evoked neurotransmitter release^2, 3^. The SNARE complex interacts with many other presynaptic proteins, such as synaptotagmin, Munc18 and complexin, which regulate exocytosis of synaptic vesicles^4, 5^. It has recently been shown that synaptotagmin 7 is essential for presynaptic plasticity^6^, although it seems difficult to explain all aspects of presynaptic plasticity only by synaptotagmin 7.

While attention has been riveted to postsynaptic plasticity for many years in the field of synaptic transmission, the mechanism of presynaptic plasticity is largely obscure, although it affects the activity of neural circuits much more drastically because neurotransmitter release is the first step of synaptic transmission. Although it has been reported that synaptic transmission may be up- or down-regulated through presynaptic modification by protein kinases, such as calcium/calmodulin-dependent protein kinase II (CaMKII), protein kinase A and PKC, and by protein phosphatases, such as calcineurin, protein phosphatase 1 (PP1) and PP2A^7, 8^, their substrates and molecular mechanisms are largely unknown. SNAP-25 is phosphorylated at the residue serine-187 by PKC^8, 9^, which is known to enhance calcium-dependent release of dopamine and acetylcholine from PC12 cells^10^ and to increase the size of the highly Ca^2+^-sensitive vesicle pool in chromaffin cells^11^. However, other studies have reported that the phosphorylation of the same residue of SNAP-25 has no influence on exocytosis of synaptic vesicles^12^. Therefore, the role of SNAP-25 phosphorylation at serine-187 is still obscure and it is unknown whether it has any effect on synaptic transmission at mammalian CNS synapses. Furthermore, there has been a heated debate whether the PKC-activator phorbol ester mediates enhancement of neurotransmitter release through phosphorylation by PKC or through its direct action on release machinery^13^. To examine these issues, we generated knock-in (KI) mice deficient in the phosphorylation by replacing serine-187 of SNAP-25 with alanine^14^ and analyzed synaptic transmission and plasticity at hippocampal CA1 synapses. In the previous paper examining this KI mouse, it has been reported that it exhibits epileptic seizures at the age of around 4 weeks and severe anxiety-like behaviors in adults^14^. It has also been shown that the mutant SNAP-25 expression of KI mice is reduced to about half of wild-type (WT) mice^14^.

In the present study, we examined electrophysiological, biochemical and morphological phenotypes of the KI mouse in great detail to elucidate physiological roles of the SNAP-25 phosphorylation in synaptic transmission and presynaptic plasticity. We found that the efficacy of basal synaptic transmission was decreased, whereas presynaptic short-term plasticity was drastically enhanced in KI mice, which became more remarkable with development in parallel with the increase of the SNAP-25 phosphorylation and was at least partially mediated by the change in synaptic vesicle dynamics. Electron-microscopy revealed the accumulation of synaptic vesicles in enlarged presynaptic terminals in KI mice. We also confirmed that these phenotypes were not caused by a decrease of SNAP-25 expression nor by an epileptic seizure. These results indicate that this phosphorylation exerts great influence on synaptic functions in the CNS, and it may regulate higher brain functions such as emotion and prevent excessive activities in the brain such as an epileptic seizure through inhibition of presynaptic plasticity.

## Results

### Basal synaptic efficacy is decreased and short-term presynaptic plasticity is enhanced in KI mice deficient in SNAP-25 phosphorylation by PKC

**Figure 1:**
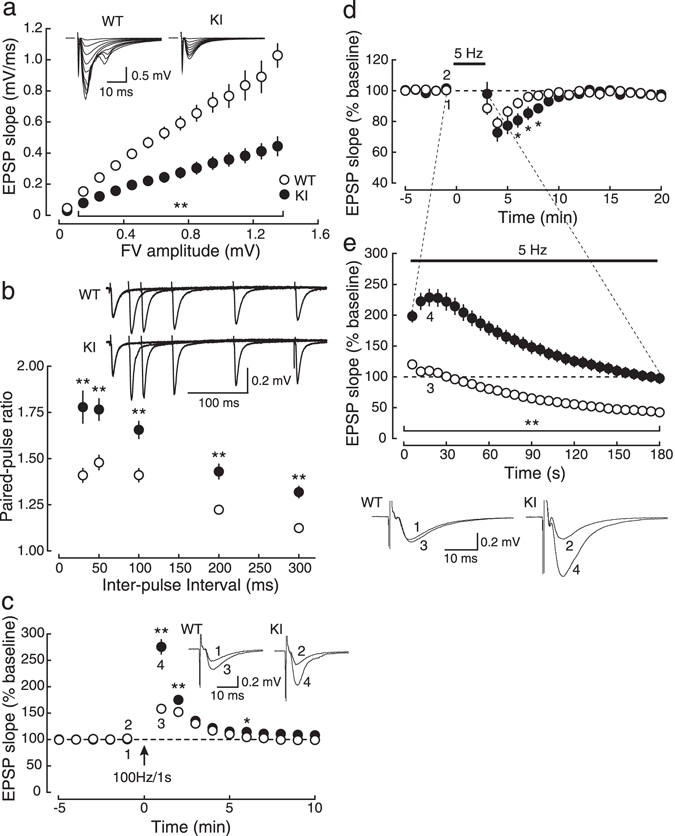
Abnormal basal synaptic transmission and presynaptic short-term plasticity in adult (9–15 weeks old) KI mice. (**a**) The input (fiber-volley amplitude)-output (EPSP slope) relationship of AMPA receptor-mediated EPSPs at the Schaffer collateral-CA1 pyramidal cell synapse in acute hippocampal slices of KI (closed circles:●, *n* = 8) and their littermate WT (open circles:○, *n* = 9) mice. The maximum initial slope of AMPA receptor-mediated EPSPs evoked with the stimulus intensities ranging from 0.9 to 5.6 V is plotted as a function of the fiber volley amplitude. Sample traces of EPSPs (averages of 10 consecutive sweeps) evoked with various stimulus strengths are shown in the inset. (**b**) PPF (the ratio of slopes of the second EPSP to those of the first EPSP) shown as a function of inter-pulse intervals in the presence of 25 μM D-APV (WT, open circles:○, *n* = 13; KI, closed circles:●, *n* = 10). In the inset, sample traces of synaptic responses evoked by paired stimuli at intervals of 30, 50, 100, 200 and 300 ms are superimposed. (**c**) The time course of PTP elicited by tetanic stimulation (100 Hz, 1 s) in the presence of 50 μM D-APV (WT, open circles:○, *n* = 12; KI, closed circles:●, *n* = 10). Sample traces of synaptic responses during the baseline and at the peak of the potentiation at the time points indicated by the numbers in the graph are shown in the inset. (**d**,**e**) After obtaining a stable baseline at 0.1 Hz at least for 20 min (**d**) (only the data of the last 5 min are shown), 5-Hz stimulation was applied for 3 min (**e**), and then the stimulus frequency was returned to 0.1 Hz (**d**, 3–20 min) (WT, open circles:○, *n* = 11; KI, closed circles:●, *n* = 10). Sample traces of synaptic responses during the baseline and 5-Hz stimulation at the time points indicated by the numbers in the graph are illustrated below the graph. For clarity, stimulus artifacts are truncated in all sample traces.

In order to elucidate the physiological role of SNAP-25 phosphorylation in the regulation of basal synaptic transmission, we first examined the input-output relationship of excitatory synaptic responses in the CA1 region of acute hippocampal slices obtained from adult mice (9–15 weeks old) using the extracellular field-potential recording technique (Fig. 1a). The slope of α-amino-3-hydroxy-5-methyl-4-isoxazolepropionic acid (AMPA) receptor-mediated excitatory postsynaptic potentials (EPSPs) (output) in response to the stimulation that evoked the same fiber volley amplitude (input) in KI mice was consistently smaller than that in WT mice, indicating that the efficacy of basal synaptic transmission in KI mice was decreased compared to WT mice.

Paired-pulse facilitation (PPF) at inter-stimulus intervals of 30, 50, 100, 200 and 300 ms was remarkably larger in KI mice (Fig. 1b), indicating that the reduced synaptic efficacy in KI mice was due to the decrease of presynaptic neurotransmitter release probability^15^. Post-tetanic potentiation (PTP) induced by tetanic stimulation (100 Hz, 1 s) in the presence of the N-methyl-D-aspartate (NMDA) receptor antagonist D-2-amino-5-phosphonovaleric acid (D-APV), which is another form of presynaptic short-term plasticity, was also enhanced in KI mice (Fig. 1c), further suggesting that presynaptic short-term plasticity was impaired in KI mice. Furthermore, synaptic responses to prolonged low-frequency stimulation (5 Hz, 3 min) in KI mice were markedly larger (*P* < 0.05 for all time points during 5-Hz stimulation) than those in WT mice (Fig. 1e), and the time constant of the recovery from transient synaptic depression after 5-Hz stimulation in KI mice (5.27 ± 0.89 min, *n* = 10) was significantly larger (*P* = 0.0323) than that in WT mice (2.89 ± 0.46 min, *n* = 11) (Fig. 1d), suggesting that the dynamics of synaptic vesicles in the presynaptic terminal was impaired in KI mice. These results suggested that the fusion process of synaptic vesicles in basal conditions as well as the dynamics of synaptic vesicles during repetitive synaptic activation was severely impaired in KI mice, which indicated that the basal neurotransmitter release probability was decreased, but the synaptic efficacy during repetitive synaptic activation was increased in KI mice.

In order to exclude the possible contribution of the postsynaptic component to presynaptic phenotypes in KI mice, we examined the expression and distribution of AMPA receptors as well as miniature excitatory postsynaptic currents (mEPSCs) in the CA1 region of the hippocampus. Immunohistochemistry of the GluA2 subunit of AMPA receptors demonstrates that GluA2 distribution is similar between the genotypes (Supplementary Fig. S1). The analysis of amplitudes of mEPSCs, which represent the smallest synaptic response at a single synapse level whose amplitude is proportional to the number of AMPA receptors on spines of the dendrite, revealed that the mEPSC amplitude distribution was indistinguishable between the genotypes (*P* = 0.9639, Kolmogorov-Smirnov test) (Supplementary Fig. S2). These results suggest that AMPA receptors are not associated with presynaptic phenotypes observed in KI mice. Interestingly, inter-event intervals are also indistinguishable between the genotypes (*P* = 0.9972, Kolmogorov-Smirnov test), suggesting that SNAP-25 phosphorylation may not be involved in spontaneous release of glutamate from synaptic vesicles (Supplementary Fig. S2).

### The number of synaptic vesicles in the presynaptic terminal is increased in KI mice

**Figure 2:**
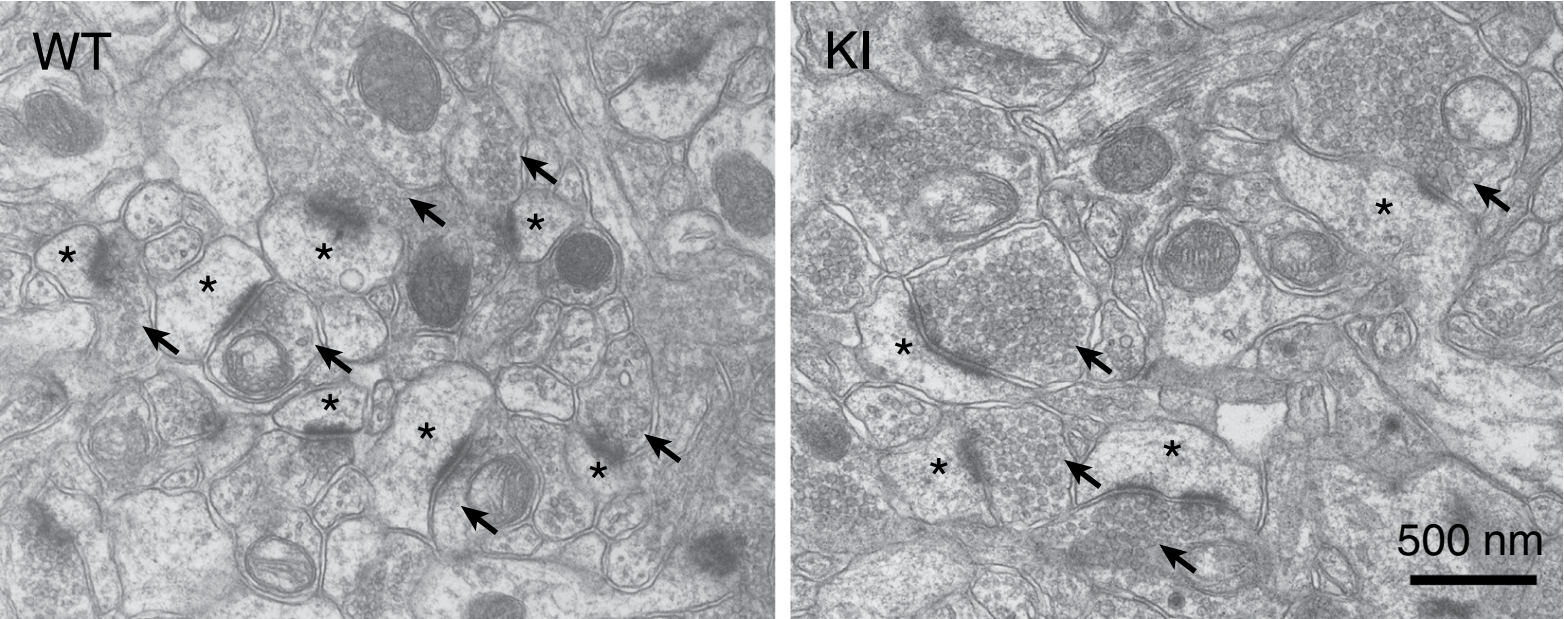
Representative electron micrographs of the hippocampal CA1 region in WT (left) and KI (right) mice. Arrows (↑) and asterisks (*) indicate excitatory presynaptic terminals and spines, respectively.

By electron microscopy, we analyzed axo-spinous synapses in the hippocampal CA1 region (Fig. 2). We found that the mean profile area of presynaptic terminals was significantly increased, whereas the density of synapses was significantly reduced in KI mice (Table 1). No genotypic differences were noted in the density of synaptic vesicles in terminals, the size of the active zone and the number of docked synaptic vesicles per active zone (Fig. 2; Table 1). These results suggested that the number of synaptic vesicles in given terminals was increased in KI mice, with no appreciable structural changes in the active zone or docked vesicles.

**Table 1:** Ultrastructural parameters of synapses examined by electron microscopy.

Parameter	Wild-type mice	SNAP-25 KI mice
Synapse density (per 100 μm^2^)	48.8 ± 2.2	32.6 ± 4.7**
Perforated synapses (% of all synapses)	5.85 ± 2.15	5.28 ± 1.99
Presynaptic terminal area (μm^2^)	0.17 ± 0.01	0.31 ± 0.06*
Synaptic vesicle density in terminals (per μm^2^)	151.2 ± 13.1	161.5 ± 37.7
Size of active zone (nm)	203.4 ± 27.9	212.9 ± 11.3
Docked synaptic vesicles (per μm active zone)	7.49 ± 1.54	7.79 ± 0.62

All data are from the distal striatum radiatum of the hippocampal CA1 region in WT and KI mice. Data are presented as the mean ± SD. *P < 0.05, **P < 0.01 (Student’s *t*-test).

### The reduction of the expression of SNAP-25 is not associated with the presynaptic phenotypes of KI mice

**Figure 3:**
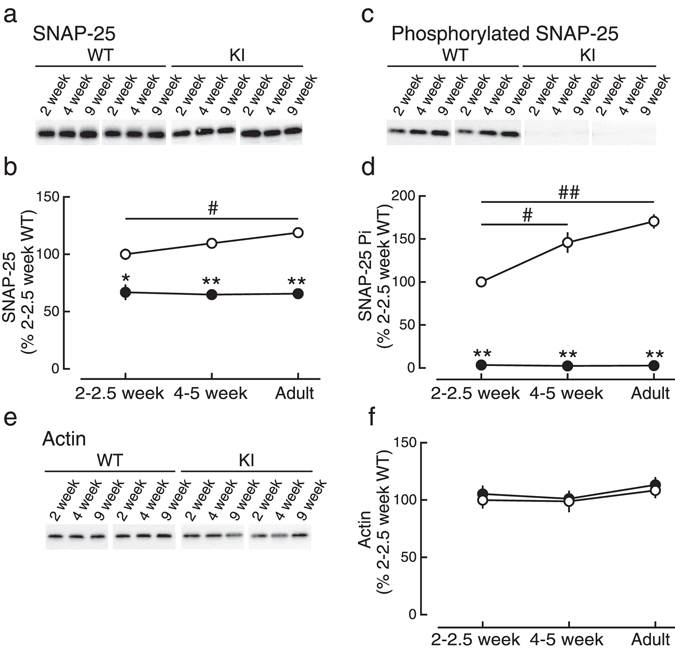
Developmental changes in SNAP-25 expression and phosphorylation in the hippocampus. (**a**,**b**) Expression levels of SNAP-25 in the hippocampus were measured by Western blot analyses. The density of the bands was normalized to that of 2–2.5-week-old WT mice. (**c**,**d**) Phosphorylation levels of SNAP-25 at the residue serine-187 in the hippocampus were measured by Western blot analyses. The density of the bands was normalized to that of 2–2.5-week-old WT mice. (**e**,**f**) Expression levels of actin in the hippocampus were measured as a control. * and ^#^ indicate significant differences between the genotypes and between the ages, respectively.

**Figure 4:**
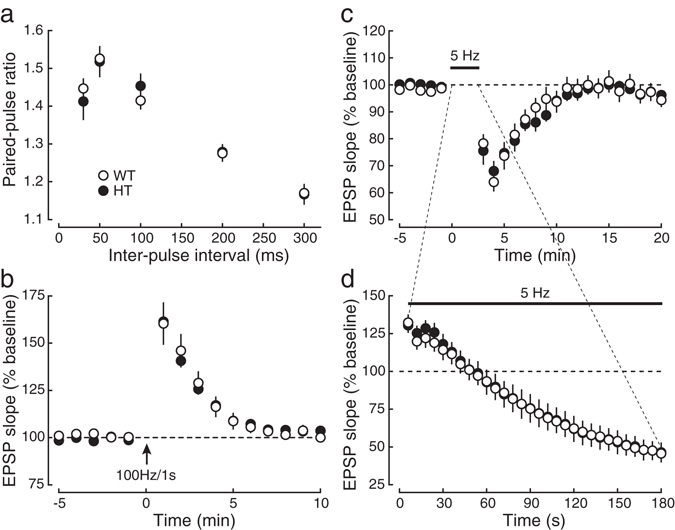
The decrease in the expression of SNAP-25 protein in KI mice is not associated with the impairment in basal synaptic transmission and presynaptic short-term plasticity. (**a**) The paired-pulse ratio was indistinguishable between HT (closed circles:●, *n* = 8) and WT mice (open circles:○, *n* = 9) at all inter-pulse intervals. (**b**) PTP induced by tetanic stimulation (100 Hz, 1 s) in the presence of 50 μM D-APV in HT mice (closed circles:●, *n* = 4) was not significantly different from that in WT mice (open circles:○, *n* = 5). (**c**,**d**) Synaptic responses during 5-Hz stimulation (**d**) and synaptic depression after the stimulation (**c**) were indistinguishable between HT (closed circles:●, *n* = 5) and WT mice (open circles:○, *n* = 6).

In KI mice, it has been reported that the expression of mutant SNAP-25, but not that of the other major synaptic proteins, is reduced to about half of WT mice^14^. We confirmed that the expression of mutant SNAP-25 was also reduced to about half in our present study (Fig. 3a,b), and, therefore, examined synaptic transmission in SNAP-25 heterozygous (HT) knockout (KO) mice^2^, in which the expression of SNAP-25 was reduced to about half of WT mice as in KI mice (data not shown). In contrast to the results in KI mice, PPF and PTP were both indistinguishable between HT and WT mice (Fig. 4a,b). Furthermore, the responses during and after 5-Hz stimulation were also unchanged in HT mice (Fig. 4c,d). These results indicated that the electrophysiological phenotypes observed in KI mice were not caused by the reduction of the expression of SNAP-25, in contrast to the previous report showing that reduced SNAP-25 alters short-term synaptic plasticity^16^.

### Epileptic seizure observed in KI mice is not related to presynaptic electrophysiological phenotypes

**Figure 5:**
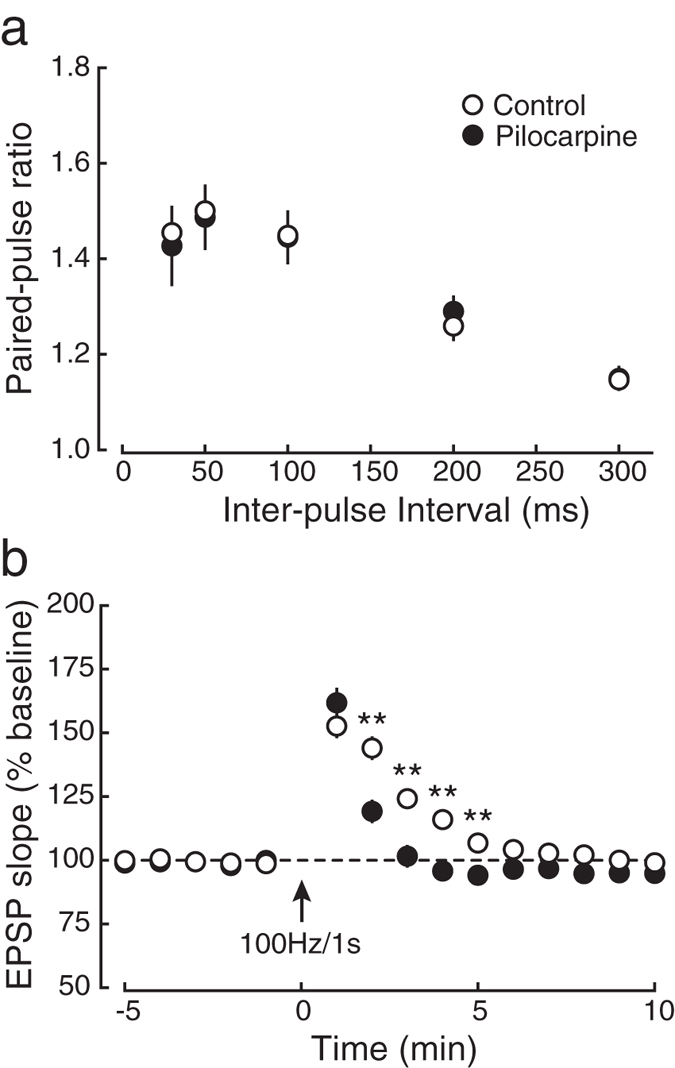
Epileptic seizures are not responsible for the phenotypes in short-term synaptic plasticity in KI mice. (**a**) The paired-pulse ratio was indistinguishable between saline-injected control mice (open circles:○, *n* = 8) and those exhibited an epileptic seizure by intraperitoneal administration of pilocarpine (closed circles:●, *n* = 8) at all inter-pulse intervals. (**b**) PTP elicited by tetanic stimulation (100 Hz, 1 s) was indistinguishable between pilocarpine-treated (closed circles:●, *n* = 8) and control mice (open circles:○, *n* = 8), although the potentiation 2 to 5 min after tetanic stimulation was significantly different between the groups.

Because almost all KI mice exhibit epileptic seizure at around 4 weeks after birth^14^, it is possible that the phenotypes demonstrated so far may have resulted from the episode of epileptic seizure. To examine this possibility, we injected the muscarinic acetylcholine receptor agonist pilocarpine intraperitoneally into normal mice, which caused generalized tonic-clonic seizure in all mice tested (see **Methods**), and analyzed PPF and PTP using hippocampal slices obtained from these pilocarpine-treated mice (Fig. 5). We failed to find any difference in PPF between pilocarpine-treated and saline-injected control mice (Fig. 5a). In PTP experiments, there was no significant difference at the first time point after tetanic stimulation between pilocarpine-treated and saline-injected control mice (Fig. 5b). Interestingly, EPSPs at the second to fifth time point after tetanic stimulation in pilocarpine-treated mice were decreased rather than increased (Fig. 5b). Therefore, the enhanced PTP in KI mice was unlikely to result from the episode of epilepsy.

### Developmental changes in SNAP-25 phosphorylation and presynaptic short-term plasticity

**Figure 6:**
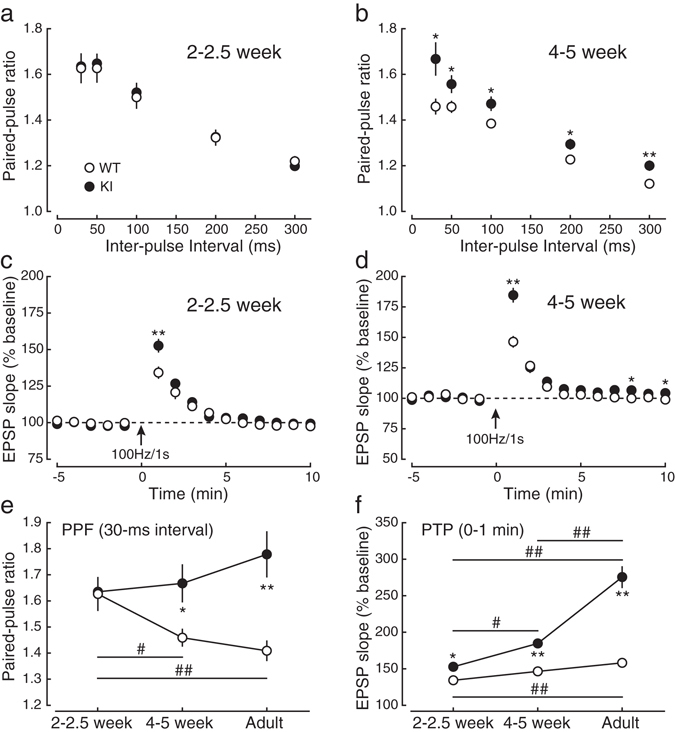
Developmental changes in presynaptic short-term plasticity. (**a**) The paired-pulse ratio was not significantly different between 2–2.5-week-old KI (closed circles:●) and WT (open circles:○) mice at any inter-pulse interval. (**b**) The paired-pulse ratio was higher in 4–5-week-old KI mice than in 4–5-week-old WT mice. (**c**) PTP induced by tetanic stimulation was larger in 2–2.5-week-old KI mice (closed circles:●) than in 2–2.5-week-old WT mice (open circles:○). (**d**) PTP in 4–5-week-old KI mice was larger compared with 4–5-week-old WT mice. (**e**) Developmental changes in PPF (at the interval of 30 ms). (**f**) Developmental changes in PTP (the first time point after tetanic stimulation). * and ^#^ indicate significant differences between the genotypes and between the ages, respectively.

We next examined the developmental changes of SNAP-25 expression and serine-187 phosphorylation (Fig. 3) as well as presynaptic short-term plasticity (Fig. 6), and compared the results between WT and KI mice. The expression levels of SNAP-25 protein was unchanged throughout the ages examined in both WT and KI mice except the difference between 2–2.5-week-old and adult WT mice (*P* = 0.025, Tukey’s test), although the level in KI mice was about half of WT mice in all ages (Fig. 3a,b) and the differences were statistically significant. In contrast, the levels of serine-187 phosphorylation of SNAP-25 in WT mice were increased with development (Fig. 3c,d), although the difference between 4–5 week-old and adult mice did not reach the significant level (*P* = 0.150), while the phosphorylation level in KI mice was below the detection level at all ages. The expression levels of actin were unchanged throughout the development in both WT and KI mice (Fig. 3e,f).

We next examined PPF and PTP at the age of 2–2.5 and 4–5 weeks (Fig. 6) and compared the results with those of adult mice (Figs 1b,c and 6e,f). At the age of 2–2.5 weeks, PPF was indistinguishable between the genotypes (Fig. 6a: *P* > 0.05 for all the intervals), whereas it was consistently larger at any inter-pulse interval in 4–5-week-old KI mice than in 4–5-week-old WT mice (Fig. 6b). We also examined PTP in 2–2.5- and 4–5-week-old mice (Fig. 6c,d). In contrast to PPF, PTP was significantly increased even at the age of 2–2.5 weeks in KI mice compared to WT mice (Fig. 6c), and the ratio of increase was much larger at the age of 4–5 weeks in KI mice (Fig. 6d). Developmental changes in PPF and PTP are summarized in Fig. 6e,f, respectively, showing that PPF was decreased with age in WT mice (Fig. 6e, open circles, the 30-ms interval: 2–2.5 week, 1.63 ± 0.06, *n* = 14; 4–5 week, 1.46 ± 0.03, *n* = 13; Adult, 1.41 ± 0.04, *n* = 13: 2–2.5 week vs. 4–5 week, *P* = 0.041; 2–2.5 week vs. Adult, *P* = 0.006; 4–5 week vs. Adult, *P* = 0.740, Tukey’s test) although there was no significant difference between 4–5-week-old and adult mice, whereas it was unchanged throughout the examined ages in KI mice (Fig. 6e, closed circles, the 30-ms interval: 2–2.5 week, 1.63 ± 0.04, *n* = 15; 4–5 week, 1.67 ± 0.07, *n* = 12; Adult, 1.78 ± 0.09, *n* = 10: 2–2.5 week vs. 4–5 week, *P* = 0.925; 2–2.5 week vs. Adult, *P* = 0.266; 4–5 week vs. Adult, *P* = 0.479). In contrast, PTP was drastically increased with age in KI mice (Fig. 6f, closed circles, 0–1 min: 2–2.5 week, 152.6 ± 4.2%, *n* = 11; 4–5 week, 184.6 ± 5.5%, *n* = 12; Adult, 275.5 ± 14.1%, *n* = 10: 2–2.5 week vs. 4–5 week, *P* = 0.028; 2–2.5 week vs. Adult, *P* < 0.001; 4–5 week vs. Adult, *P* < 0.001), whereas it was increased only slightly but significantly in WT mice when compared between 2–2.5-week-old and adult mice (Fig. 6f, open circles, 0–1 min: 2–2.5 week, 134.2 ± 3.7%, *n* = 9; 4–5 week, 146.3 ± 4.2%, *n* = 11; Adult, 158.0 ± 4.8%, *n* = 12: 2–2.5 week vs. 4–5 week, *P* = 0.163; 2–2.5 week vs. Adult, *P* = 0.002; 4–5 week vs. Adult, *P* = 0.142). PPF was significantly different between WT and KI mice in 4–5-week-old (*P* = 0.033, Bonferroni’s test) and adult (*P* < 0.001) mice, although it was indistinguishable in 2–2.5-week-old mice (*P* = 1.000). PTP was significantly different between WT and KI mice at all ages (2–2.5 week, *P* = 0.015; 4–5 week, *P* < 0.001; Adult, *P* < 0.001). These results indicated that PPF and PTP were developmentally changed, but were differentially regulated by the serine-187 phosphorylation of SNAP-25. Therefore, it is concluded that the serine-187 phosphorylation of SNAP-25 inhibits presynaptic plasticity in an age-dependent manner in normal animals.

### The refilling rate of synaptic vesicles is faster in KI mice

**Figure 7:**
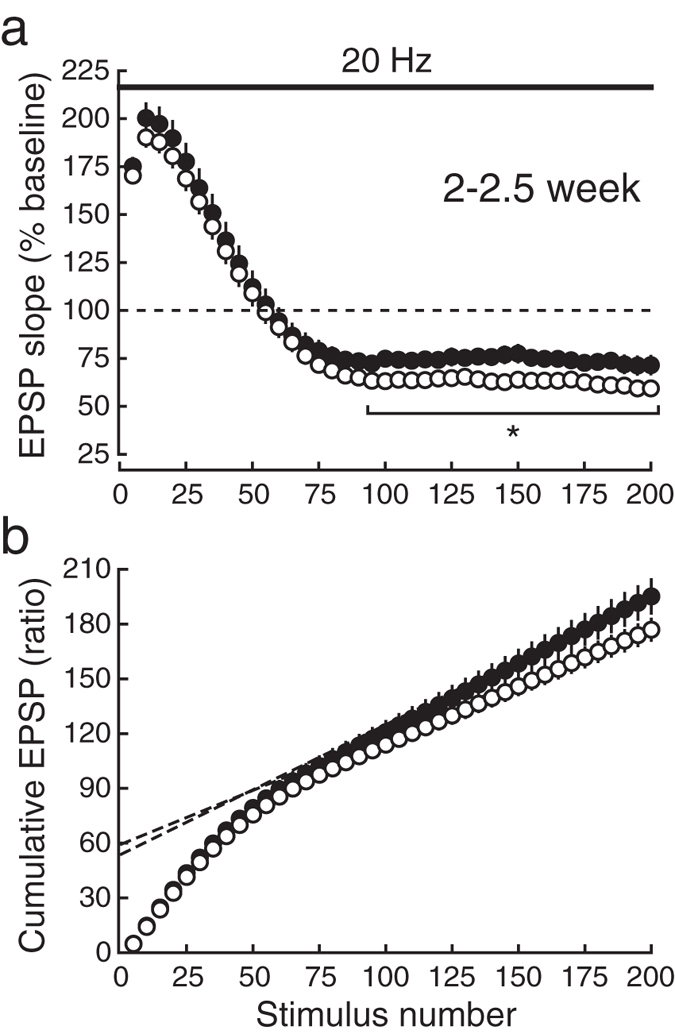
Synaptic responses during higher-frequency repetitive stimulation in juvenile mice. (**a**) The time course of EPSPs during 20-Hz trains in CA1 synapses in juvenile WT (open circles:○) and KI (closed circles:●) mice. During 20-Hz stimulation, the depression of EPSPs in the early phase was similar in WT and KI mice. In contrast, the steady-state responses in the late phase were significantly larger in KI mice compared with WT mice. (**b**) The cumulative plot of EPSP amplitudes during 20-Hz trains, which were normalized to those of the baseline EPSP, is shown. The broken lines represent the regression lines fitted to the last 10 points (from the 191st to the 200th pulse) of EPSPs in KI (closed circles:●) and WT (open circles:○) mice.

To estimate the size of readily-releasable pools and the refilling rate of synaptic vesicles from the reserve pool to the readily-releasable pool at the age of 2–2.5 weeks, we examined synaptic responses during 20-Hz stimulation^17^ (Fig. 7). At this age, PPF was indistinguishable between the genotypes, suggesting that the initial release probability in KI mice was comparable to that in WT mice, which is a prerequisite for the following analyses of the pool size and refilling rate of synaptic vesicles^18, 19^. We gave prolonged 20-Hz stimulation (Fig. 7a), and plotted the cumulative EPSP slope values and the regression line was fitted to the points from the 191st to 200th stimulus number (Fig. 7b). When the initial release probability is unchanged, the relative size of readily releasable pool and the refilling rate of synaptic vesicles to the readily releasable pool can be compared between the genotypes by measuring the intercept of the regression line with the Y-axis and the slope of the regression line, respectively^18, 19^. This analysis indicated that the pool size was not different between the genotypes (intercept: WT, 59.42 ± 4.20, *n* = 18; KI, 53.95 ± 3.88, *n* = 15, *P* = 0.3538), but the refilling rate in KI mice was faster than that in WT mice (slope: WT, 0.59 ± 0.03; KI, 0.71 ± 0.05, *P* = 0.0382). Therefore, even soon after birth, the phosphorylation of SNAP-25 was involved in the regulation of dynamics of synaptic vesicles in the presynaptic terminal, although the size of readily-releasable pool was indistinguishable between the genotypes.

## Discussion

In this study, we analyzed KI mice deficient in phosphorylation of serine-187 of SNAP-25, which is preferentially phosphorylated by PKC^8, 9^, to elucidate the roles of this phosphorylation in presynaptic functions. We found that KI mice exhibited several abnormalities of excitatory synaptic transmission in the CA1 region of acute hippocampal slices, including the decreased basal synaptic transmission, the increased paired-pulse ratio indicative of the reduced probability of neurotransmitter release, the increased PTP and the enhanced synaptic transmission during 5-Hz repetitive stimulation. Furthermore, the size of readily-releasable pool of synaptic vesicles was unchanged but the refilling rate of synaptic vesicles was faster in KI mice. All of these phenotypes indicate for the first time that the phosphorylation of SNAP-25 is closely related to the regulation of presynaptic functions in physiological conditions, although the role of the phosphorylation in exocytosis was examined in chromaffin and PC12 cells^20, 21^. Interestingly, our data also indicate that developmental changes in release probability and presynaptic plasticity are regulated by the SNAP-25 phosphorylation.

The significance of the phosphorylation of presynaptic proteins by the phorbol ester, such as diacylglycerol, in neurotransmitter release has been the focus of an active debate for many years. In the earliest study, phorbol esters were thought to act on postsynaptic PKC, resulting in postsynaptic potentiation^22^. It has, then, been shown that phorbol esters enhance Ca^2+^-triggered exocytosis from adrenal chromaffin cells^23^ and regulate the size of the readily releasable pool of synaptic vesicles in the hippocampus^24^. However, several lines of evidence indicate that the effect of phorbol esters on synaptic transmission is not necessarily mediated by the phosphorylation by PKC and that phorbol esters might directly affect presynaptic molecules other than SNARE proteins^13, 25, 26^. Therefore, it is still unclear whether phosphorylation by PKC is involved in neurotransmitter release and presynaptic facilitation. In our present study, we have demonstrated, using a genetic approach, that excitatory synaptic transmission in the CA1 region of the hippocampus is enhanced, but presynaptic plasticity is strongly inhibited by the phosphorylation by PKC. Therefore, previously-reported presynaptic enhancement caused by phorbol esters is quite likely induced by the phosphorylation of SNAP-25 mediated by PKC, although phosphorylation-independent regulation of presynaptic release machinery by phorbol esters cannot be excluded^13^.

It is also known that SNAP-25 phosphorylation increases its association with syntaxin^11^ in chromaffin cells, another SNARE protein, which facilitates the formation of the SNARE complex consisting of presynaptic proteins including synaptobrevin and synaptotagmin that directly regulates exocytosis of synaptic vesicles from the presynaptic terminal. SNAP-25 is also reported to interact with synaptotagmin in a Ca^2+^-dependent manner in PC12 cells, which is essential for Ca^2+^-triggered exocytosis^27^, and with P/Q-type Ca^2+^ channels *in vitro* ^28^ and N-type Ca^2+^ channels in chromaffin cells^29^, both of which are also required for evoked synaptic transmission. It is also shown that activity-dependent phosphorylation of serine-187 of SNAP-25 is required for negative modulation of voltage-gated Ca^2+^ channels in hippocampal slices^30^. Therefore, it is possible that the phosphorylation of SNAP-25 increases synaptic efficacy through modification of SNARE proteins and/or Ca^2+^ channels. Further investigation of the role of SNAP-25 phosphorylation in the SNARE complex at synapses would provide novel insights into the molecular mechanism of the modulation of presynaptic functions in the brain.

It has been thought that synaptic facilitation of the second response in PPF is induced by the residual Ca^2+^ resulting from the first synaptic activation^31^. The molecular target of the increased Ca^2+^ for PPF has not definitely been identified, although the characteristic properties of PPF have been clarified^15^. However, recently, synaptotagmin 7 was proposed as a mediator of presynaptic facilitation such as PPF and frequency facilitation induced by repetitive synaptic activation^6^. Whereas synaptotagmin 7 is not associated with basal synaptic transmission^6^, the mutant SNAP-25 lacking the PKC phosphorylation site decreased basal synaptic efficacy as shown in our present study. Furthermore, our study has demonstrated that the lack of phosphorylation of SNAP-25 by PKC drastically increases PPF, indicating that the phosphorylation strongly inhibits PPF in normal animals. Therefore, SNAP-25 exhibits more wide-ranging roles in the presynaptic regulation, and synaptotagmin 7 may play a role downstream of SNAP-25. It would be a very interesting and important issue in future studies to examine the relationship between synaptotagmin 7 and SNAP-25 phosphorylation in terms of presynaptic plasticity.

PTP is also one of the well-known presynaptic plasticity in the CNS; however, its expression mechanism has been largely unknown, although it has been shown that it is expressed presynaptically^32^. It has been demonstrated that PTP is mediated by activity-dependent activation of PKC in the presynaptic terminal of CA1 synapses in hippocampal slice cultures^33^ and that enhanced Ca^2+^ sensitivity of the fusion of synaptic vesicles mediated by PKC is associated with PTP in the presynaptic terminal of synapses in the calyx of Held^34^. However, the target molecule of PKC is totally unknown, and moreover, it is still to be determined whether the phosphorylation by PKC itself is responsible for these effects on PTP. In our present study, we have clearly shown that phosphorylation of SNAP-25 by PKC is involved in the expression of PTP. However, although the previous reports demonstrated that PKC is required for the PTP expression, our present study has indicated that the magnitude of PTP is markedly increased in KI mice lacking the phosphorylation by PKC, indicating that PTP is inhibited by PKC, as opposed to the previous reports. Taken together, our results strongly suggest that SNAP-25 dynamically regulates presynaptic release processes and plasticity through its phosphorylation.

Synaptic modification by higher-frequency repetitive activation of synapses, such as that induced by 5-Hz stimulation used in this study (Fig. 1), is sensitive to presynaptic regulatory molecules. We have shown in the previous study^35^ that the synaptic vesicle-associated Ca^2+^-binding protein Doc2α inhibits synaptic responses during 5-Hz stimulation but that it has no effect on PPF or PTP, suggesting that Doc2α regulates synaptic vesicle dynamics in the presynaptic terminal but has no direct effect on the release process. We have also demonstrated that the presynaptic active zone protein CAST enhances neurotransmitter release but has no effect on synaptic responses during 5-Hz stimulation, whereas it facilitates the recovery from the depression after 5-Hz stimulation^36^, suggesting that CAST modulates both neurotransmitter release and synaptic vesicle dynamics. These results indicate that analysis of the behavior of synaptic transmission during and after 5-Hz repetitive stimulation is useful for evaluating the properties of synaptic vesicle dynamics, and our present results suggest that the phosphorylation of SNAP-25 regulates both neurotransmitter release and synaptic vesicle dynamics. Furthermore, the results of the experiments in which synaptic responses to 20-Hz stimulation (Fig. 7) were examined in juvenile mice suggest that the size of readily-releasable pools of synaptic vesicles is not different between the genotypes but that the rate of refilling of synaptic vesicles from reserve pools to readily-releasable pools is faster in KI mice.

Although it seems to be reasonable that SNAP-25 phosphorylation regulates neurotransmitter release because SNAP-25 is mainly localized in the membrane of presynaptic terminals, it is a rather unexpected result that SNAP-25 phosphorylation also modulates synaptic vesicle dynamics. However, it has been shown that SNAP-25 is also localized on synaptic vesicles although not abundantly^37^, and this minor component of SNAP-25 may be sufficient enough for the regulation of synaptic vesicle dynamics. Alternatively, endocytosis of synaptic vesicles in the terminal may be regulated by the phosphorylation of SNAP-25 localized in the terminal membrane, which could affect the dynamics of endocytosed synaptic vesicles. Furthermore, another presynaptic molecule may indirectly mediate the modulation of synaptic vesicle dynamics. For instance, Snapin, which was originally identified as a SNAP-25-binding protein, is known to enhance the interaction of synaptotagmin and SNAP-25 and to modulate synaptic vesicle trafficking and distribution in chromaffin cells and hippocampal neurons^38, 39^. If the binding of SNAP-25 with Snapin is regulated by SNAP-25 phosphorylation, synaptic vesicle dynamics can be changed by Snapin depending on the level of SNAP-25 phosphorylation. In addition, it is interesting to note that the size of readily-releasable pools of synaptic vesicles is regulated by PKA-dependent phosphorylation of SNAP-25 in chromaffin cells^40^.

Although some types of central synapses, such as those in layer 2/3 and layer 5 pyramidal cells in the cerebral cortex^41^ and temporoammonic-CA1 synapses in the hippocampus^42^, seem to exhibit a developmental decrease in neurotransmitter release probability, most synapses show an increase in the probability during development^42–44^. In our present study, we confirmed that release probability was increased during development in WT mice as evidenced by the decrease in PPF in parallel with development (Fig. 6e). The level of phosphorylation of serine-187 of SNAP-25 was increased during development^45^ similarly to PPF. However, in KI mice, PPF remained unchanged during development, strongly suggesting that release probability was constant throughout the ages examined and SNAP-25 phosphorylation was responsible for the observed developmental change in release probability and PPF.

Since SNAP-25 phosphorylation at serine-187 was changed with age in WT mice but the phosphorylation was absent throughout development in KI mice, phosphorylation-dependent interaction of SNAP-25 with other presynaptic molecules may be related to PPF and PTP and functions of other presynaptic molecules including SNARE proteins may also change in an age-dependent manner. In WT mice, this interaction may be changed by phosphorylated SNAP-25, which may suppress presynaptic plasticity, but in KI mice, synaptic transmission may be released from this inhibition, causing such drastic increases in PPF and PTP. It is also possible that the KI mutation may affect SNARE complex assembly, resulting in the changes of neurotransmitter release and presynaptic plasticity. It would be interesting to examine these issues in future studies.

Neurotransmitter release evaluated by PPF is modulated by SNAP-25 phosphorylation only at the ages older than 4–5 weeks after birth (Fig. 6e), whereas the dynamics of synaptic vesicles is sensitive to the change in phosphorylation even at the age of 2 weeks (Figs 6f and 7). Therefore, neurotransmitter release and synaptic vesicle dynamics are likely to be regulated by mutually different mechanisms with different time courses. Although the release probability at the age of 2–2.5 weeks seems to be insensitive to SNAP-25 phosphorylation, the rate of refilling of synaptic vesicles is increased by the phosphorylation.

We have reported in the previous study that this KI mouse exhibits epilepsy^14, 46^. Although the release probability of neurotransmitters is lower in KI mice, synaptic responses are dramatically enhanced during and after high-frequency stimulation, which would overwhelm the decreased release probability and may cause over-activation of neural circuits, resulting in epileptic seizure in KI mice. Interestingly, it has also been reported that the phosphorylation of SNAP-25 is increased by neural activities^47^. Therefore, the phosphorylation of SNAP-25 may serve as a brake on abnormal excessive synaptic activities.

This KI mouse also exhibits marked anxiety-like behaviors^14^. Many neuropsychiatric diseases, including schizophrenia and depression, are accompanied by anxiety disorders. It has been shown in many previous papers^48–50^ that SNAP-25 is involved in psychiatric disorders, such as schizophrenia, attention-deficit/hyperactivity disorder and autism spectrum disorders, in mice and humans and that stress increases the SNAP-25 phosphorylation^51^. Therefore, the phosphorylation of SNAP-25 would be a promising target for developing novel therapeutics for mental disorders.

## Methods

This research was approved by the Animal Care and Experimentation Committees of University of Tokyo, Kitasato University and Hokkaido University, and all experiments were performed according to the guidelines laid down by the Committee of each institution.

### Mice

Mutant mice in which serine-187 of SNAP-25 was replaced with alanine were generated as previously reported^14^. E14TG2a mouse embryonic stem cells were used to generate mutant mice, which were backcrossed into C57BL/6 N mice for 13 generations, and these mice were used for all the experiments in this paper. Homozygous KI and their littermate WT mice were obtained by *in-vitro* fertilization using ICR mice as foster mothers. Heterozygous SNAP-25 KO and their littermate WT mice were obtained by crossing of heterozygous mice. In this study, we used a minimum number of mice that were required to draw the conclusions and tried to minimize their suffering as much as possible.

### Electrophysiological analyses

Mice were deeply anesthetized with halothane and decapitated. The brains were quickly removed and hippocampal slices (400 µm in thickness) were prepared acutely from mice (WT and KI: 2–2.5, 4–5 or 9–15 weeks old; heterozygous KO and WT: 9–14 weeks old) with a tissue slicer (Leica Microsystems, Wetzlar, Germany) using standard procedures and solutions^52, 53^ and placed in an interface-type holding chamber for at least 1 h. The extracellular solution contained (in mM): 119 NaCl, 2.5 KCl, 1.3 MgSO_4_, 2.5 CaCl_2_, 1.0 NaH_2_PO_4_, 26.2 NaHCO_3_ and 11 glucose. A single slice was then transferred to a submersion-type recording chamber continuously perfused with the extracellular solution saturated with 95% O_2_ and 5% CO_2_ at the rate of 1.5–2.0 ml/min. Synaptic responses were recorded using an Axopatch-1D amplifier (Molecular Devices, Sunnyvale, CA, USA), and the signal was filtered at 1 kHz, digitized at 10 kHz with Digidata 1322 A (Molecular Devices) and stored on a personal computer. Extracellular field-potential recordings were made in the stratum radiatum of the CA1 region using a glass electrode filled with 3 M NaCl. All the experiments were performed at 24–26 °C in the presence of 100 μM picrotoxin to block GABA_A_ receptor-mediated inhibitory synaptic responses. To exclude epileptiform activity from the CA3 region, a cut was made between the CA1 and CA3 regions. To evoke synaptic responses, Schaffer collateral/commissural fibers were stimulated at 0.1 Hz with a bipolar tungsten stimulating electrode placed in the stratum radiatum. In extracellular field-potential recordings, the stimulus strength was adjusted to obtain EPSPs with the slope value between 0.10 and 0.15 mV/ms, except for the experiments examining input-output relationships. For examining input-output relationships, a low concentration of 6-cyano-7-nitroquinoxaline-2,3-dione (CNQX, 1 μM), a non-NMDA receptor antagonist, was present to partially block AMPA receptor-mediated synaptic responses, which reduced the nonlinear summation of EPSPs and enabled more accurate measurements of fiber volley amplitudes and EPSP slopes. These experiments were performed in the presence of 25 μM D-APV to block NMDA receptor-mediated synaptic responses. PPF was induced by two consecutive stimuli delivered at inter-pulse intervals of 30, 50, 100, 200 and 300 ms in the presence of 25 μM D-APV. The paired-pulse ratio was calculated by dividing the slope value of the 2nd EPSP by that of the 1st EPSP. PTP was elicited by tetanic stimulation (100 Hz, 1 s) of afferent fibers in the presence of 50 μM D-APV. When synaptic responses to prolonged repetitive synaptic activation were examined, 900 pulses at 5 Hz or 200 pulses at 20 Hz were applied to afferent fibers in the presence of 50 μM D-APV.

### Electron microscopy

Under deep pentobarbital anesthesia (100 mg/kg body weight), 3 WT and 3 KI mice at 2 months of age were perfused transcardially with 2% paraformaldehyde/2% glutaraldehyde in 0.1 M sodium phosphate buffer (pH 7.4). Hippocampal tissues were further postfixed with 2% osmium tetroxide in 0.1 M sodium phosphate buffer (pH 7.4) for 2 h, stained with 2% uranyl acetate for 1 h and embedded in Epon 812 resin (Nissin EM, Tokyo, Japan). Ultrathin sections (70 nm in thickness) were prepared with an Ultramicrotome (ULTRACUT, Leica, Nussloch, Germany). For quantitative analyses of morphological features at axo-spinous asymmetrical synapses in the distal striatum radiatum of the hippocampal CA1 region, electron micrographs were taken with an H-7100 electron microscope (Hitachi, Tokyo, Japan). The presynaptic terminal area, length of active zone, percentage of perforated synapses, and the densities of synapses, synaptic vesicles and docked synaptic vesicles were measured by ImageJ software (National Institutes of Health, Bethesda, MD, USA). The vesicles that were located within less than 10 nm from the presynaptic membrane were defined as docked synaptic vesicles.

### Biochemical analyses

The hippocampus was removed from mice aged 2, 4 and 9 weeks euthanized by cervical dislocation and homogenized in 0.25 ml sodium dodecyl sulfate (SDS) sample buffer (70 mM SDS, 3.3% (w/v) glycerol, 0.03 mM bromophenol blue (BPB) and 125 mM Tris-HCl containing PhosStop (Roche, Tokyo, Japan); pH 6.8) by an ultrasonicator. The samples were boiled and their protein concentrations in all the samples were determined using the BCA protein assay kit (Pierce, Rockford, IL, USA). Each protein sample (0.2–5 μg) was separated by SDS-polyacrylamide gel electrophoresis (PAGE) with an acrylamide gel (15%), and then, transferred electrophoretically onto polyvinylidene difluoride membranes (Immobilon: Millipore, Billerica, MA, USA) with a semi-dry transblotting apparatus (BIO-RAD Laboratories, Hercules, CA, USA). The membrane was incubated for 20 min at room temperature in Blocking One (Nacalai, Kyoto, Japan), and then, incubated for 24 h at 4 °C with a monoclonal anti-SNAP-25 (ref. 10) or a monoclonal anti-phospho-serine-187-SNAP-25 primary antibody in Tris-buffered saline with Tween 20 (TBST: 0.05% (w/v) Tween 20, 150 mM NaCl and 25 mM Tris-HCl; pH 7.5). The monoclonal anti-phospho-serine-187-SNAP-25 antibody was obtained by immunizing mice (BALB/c: CLEA, Tokyo, Japan) with a synthetic phosphopeptide (MEKADS[Pi]NKTRI: residues 182–192 of mouse SNAP-25) conjugated to keyhole limpet hemocyanin^54^. Mice spleen cells with high titers and SP2 myeloma cells were used to generate hybridomas secreting the antibody. After the incubation of the membrane with the antibody, it was washed in the TBST and incubated for 1 h at room temperature in the TBST containing a horseradish peroxidase (HRP)-rabbit anti-mouse IgG secondary antibody. After washing in TBST, immunoreacted protein bands were visualized and quantitated by chemiluminescence using Super Signal West Pico Substrate or Super Signal West Femto Maximum Sensitivity Substrate (Thermo Fisher Scientific, Waltham, MA, USA) and a luminescence image analyzer with an electronically-cooled CCD camera (LAS-4000, Fujifilm, Tokyo, Japan). Image Quant TL (Fuji Photo Film Co., Tokyo, Japan) was used for image quantification of the bands. Three mice of each genotype were used for this analysis and two samples from a single mouse were measured. The values are normalized to those of 2-week-old C57BL/6 N mice.

### Induction of epileptic seizures

To induce sustained epileptic seizures, 8-week-old male C57BL/6 N mice (CLEA) were injected intraperitoneally with pilocarpine (280–300 mg/kg in 0.9% NaCl) as described previously^55^. To minimize peripheral side effects of pilocarpine, mice were injected intraperitoneally with scopolamine methyl nitrate (2 mg/kg) 30 min prior to pilocarpine administration. Control mice were treated with scopolamine methyl nitrate alone. Seizures were classified according to the previously-published reports^56, 57^ with slight modifications^55^. Status epilepticus was defined as sustained constant motor seizures of stage 3 or greater, which was terminated by injection of 30–40 mg/kg pentobarbital 4.5 h after the onset of status epilepticus. After status epilepticus, all animals were given soaked rodent food and 2 ml of Lactec D subcutaneously. Of pilocarpine-treated mice, only those that reached stage 6 at least once and exhibited status epilepticus (stage 3.5) for 4.5 h^55^ were used for this study. The experiments were performed 1 to 4 weeks after the administration of pilocarpine.

### Drugs

Picrotoxin, pilocarpine, scopolamine methyl nitrate and pentobarbital were purchased from Sigma-Aldrich (St. Louis, MO, USA), Lactec D from Otsuka Pharmaceutical Factory (Tokushima, Japan), TTX from Sankyo (Osaka, Japan), D-APV and CNQX from Tocris Bioscience (Avonmouth, U.K.) and halothane from Takeda Pharmaceutical Company Limited (Osaka, Japan).

### Statistical analysis

All values are expressed as the mean ± SEM, unless otherwise stated. Student’s *t*-test was used to determine whether there was a significant difference (*P* < 0.05) in the mean between two sets of data, unless otherwise stated. The data on the developmental changes in PPF and PTP (Fig. 6e,f) were analyzed using two-way ANOVA with Bonferroni or Tukey *post-hoc* tests. Statistical analysis of the biochemical experiments was performed by using one-way ANOVA followed by Tukey-Kramer’s honestly significant difference *post-hoc* tests. *^,#^ *P* < 0.05, **^,*##*^ *P* < 0.01, ***^,###^ *P* < 0.001. Where any mark (* or ^#^) is not shown in the figures, there was no significant difference (*P* > 0.05).

## Electronic supplementary material

Supplementary Information
