# Supplementary material for: SNAP-25 phosphorylation at Ser187 regulates synaptic facilitation and short-term plasticity in an age-dependent manner
Source: Sci Rep. 2017 Aug 11;7:7996. doi: 10.1038/s41598-017-08237-x (PMC5554206; doi:10.1038/s41598-017-08237-x)
Supplement: Supplementary file 1 — Supplementary Information [file 41598_2017_8237_MOESM1_ESM.pdf]

Supplementary Information

SNAP-25 phosphorylation at Ser187 regulates synaptic facilitation and short-term plasticity in an age-dependent manner

Norikazu Katayama[1], Saori Yamamori[2], Masahiro Fukaya[3,4], Shizuka Kobayashi[1], Masahiko Watanabe[4], Masami Takahashi[2] & Toshiya Manabe[1]

[1]Division of Neuronal Network, Department of Basic Medical Sciences, Institute of Medical Science, University of Tokyo, Tokyo, 108-8639, Japan.

[2]Department of Biochemistry, Kitasato University School of Medicine, Sagamihara, 252-0374, Japan.

[3]Department of Anatomy, Kitasato University School of Medicine, Sagamihara, 252-0374, Japan.

[4]Department of Anatomy, Hokkaido University Graduate School of Medicine, Sapporo, 060-8638, Japan.

Correspondence and requests of materials should be addressed to T.M. (email: tmanabe-tky@umin.ac.jp).

Supplementary Methods

Immunohistochemistry. Two-week-old mice were deeply anesthetized with isoflurane and perfused transcardially with 4% paraformaldehyde, 15 % v/v picric acid, and 100 mM phosphate buffer (pH 7.4) for 10 min with a rate of 5 ml/min. Brains were then removed from the calvaria, post-fixed overnight at 4°C in 4% paraformaldehyde, processed for paraffin embedding, and cut sagittally into 3 μm-thick sections. The sections were first treated with 1 mg/ml pepsin (Dako Japan, Tokyo, Japan) in 0.2 M HCl for 10 min at 37°C. Endogenous peroxidase activity was inhibited by incubating the sections in 3% hydrogen peroxide/calcium- and magnesium-free phosphate-buffered saline for 15 min. The sections were then immunohistochemically stained using the indirect method with a mouse anti-GluA2 6C4 antibody (BD Bioscience, Tokyo, Japan) at 10 μg/ml and Nichirei-Histofine Simple Stain Max PO(M) kits (Nichirei, Tokyo, Japan), and the immune complexes were detected with 3,3-diaminobenzidine (DAB) by using Liquid DAB+ Substrate Chromogen System (Dako Japan).

Analysis of miniature excitatory postsynaptic currents (mEPSCs). Almost the same procedures as those of field-potential recordings were used for the analysis of mEPSCs using whole-cell patch-clamp recordings. For whole-cell voltage-clamp

recordings, the patch pipette (pipette resistance: 3-5 MΩ) was filled with the internal solution containing (in mM): Cs-gluconate, 122.5; CsCl, 17.5; HEPES, 10; EGTA, 0.2; NaCl, 8; Mg-ATP, 2; Na_3-GTP, 0.3 (pH 7.2; 290-310 mOsm) and the external solution contained 1 μM tetrodotoxin (TTX) to block action potentials. mEPSCs were recorded from CA1 pyramidal cells at -80 mV and analyzed using a computer program for semiautomatic detection (Mini Analysis 6.0.7, Synaptosoft, Decatur, GA, USA). Kolmogorov-Smirnov test was used to examine whether there was significant difference in the distribution of amplitudes and inter-event intervals of mEPSCs between the genotypes.

Supplementary Figure Legends

Supplementary Figure S1. Immunostaining of the GluA2 subunit of AMPA receptors in the CA1 region of the hippocampus of 2-week-old wild-type (WT) and knock-in (KI) mice.

Supplementary Figure S2. Analysis of mEPSCs in the CA1 region of the hippocampus. (**a**) Sample traces of spontaneous mEPSCs recorded from CA1 pyramidal cells. Upper two traces are raw data of mEPSCs and lower two traces are averaged

mEPSCs. (**b**) Cumulative histogram of mEPSC amplitudes. There was no statistically significant difference (*P* = 0.9639) in the distribution between WT (*n* = 26) and KI (*n* = 19) mice. (**c**) Cumulative histogram of inter-event intervals of mEPSCs. There was no statistically significant difference (*P* = 0.9972) in the distribution between WT and KI mice.

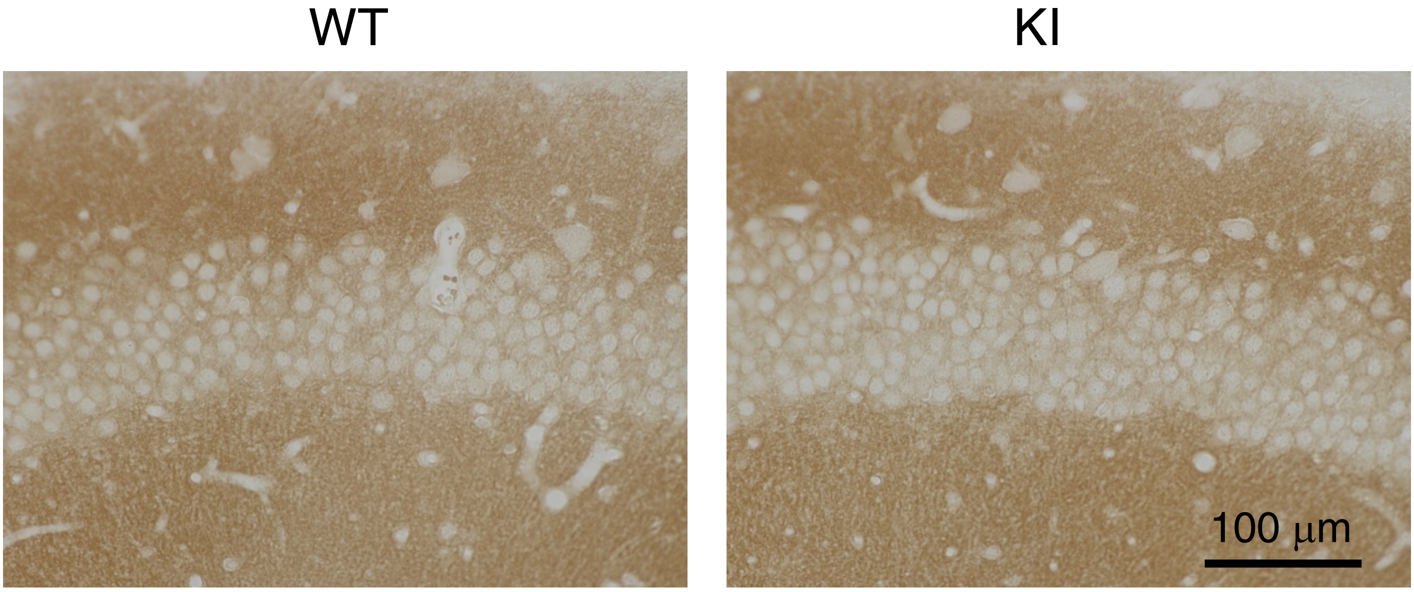

Figure S1

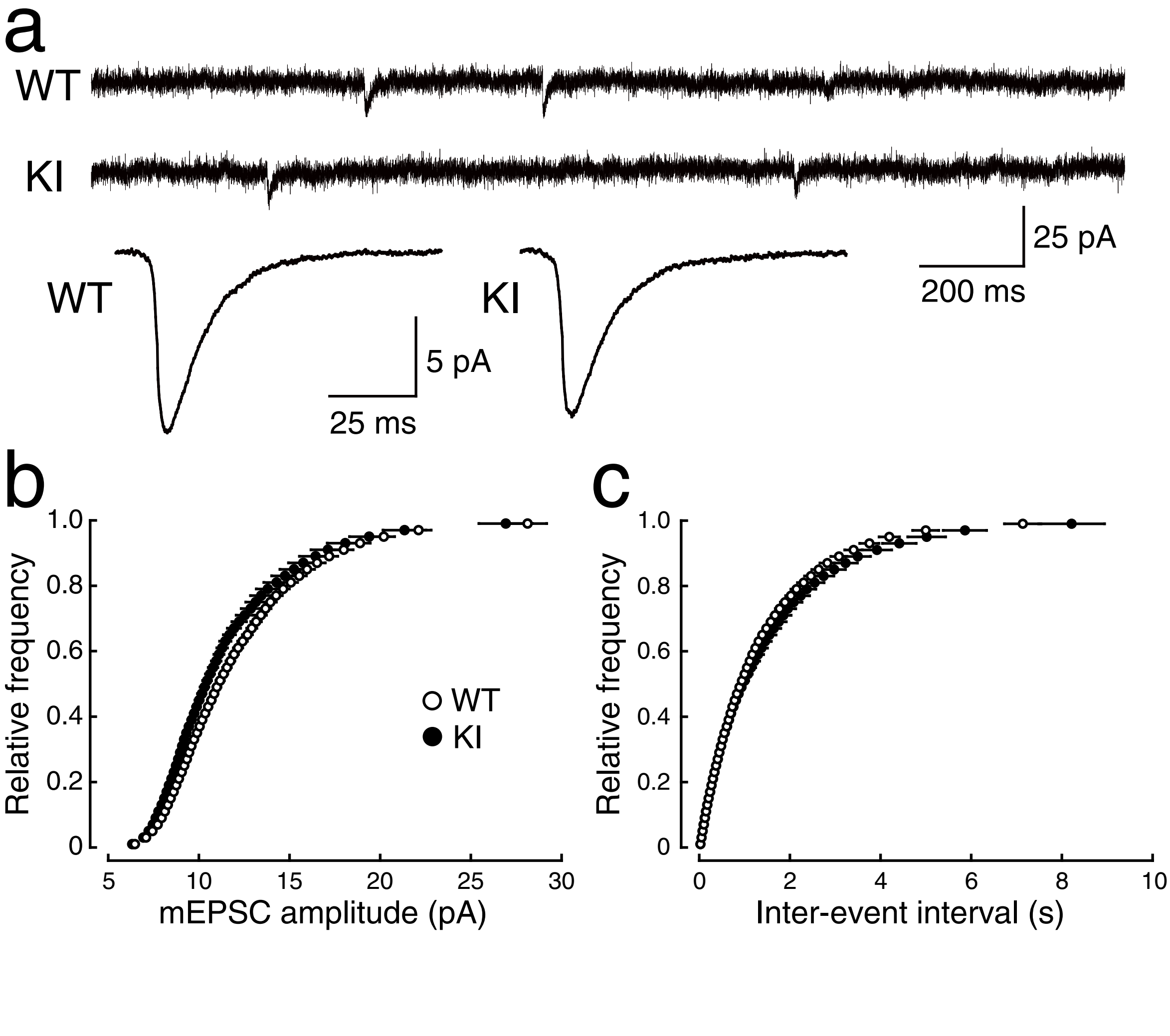
a
WT
KI
25 pA
200 ms
WT
KI
5 pA
25 ms
b
Relative frequency
1.0
0.8
0.6
0.4
0.2
0
5
10
15
20
25
30
mEPSC amplitude (pA)
WT
KI
c
Relative frequency
1.0
0.8
0.6
0.4
0.2
0
0
2
4
6
8
10
Inter-event interval (s)

Figure S2
